# Explainable SHAP-XGBoost models for identifying important social factors associated with the atherosclerotic cardiovascular disease risk score using the LASSO feature selection technique

**DOI:** 10.4178/epih.e2025052

**Published:** 2025-09-10

**Authors:** Jungtae Choi, Jooeun Jeon, Hyoeun An, Hyeon Chang Kim

**Affiliations:** 1 Department of Social Welfare, Sungkonghoe University, Seoul, Korea; 2 Center for Intelligent Digital Therapeutic Devices, Sungkyunkwan University, Seoul, Korea; 3 Department of Preventive Medicine, Yonsei University College of Medicine, Seoul, Korea; 4 Department of Public Health, Yonsei University Graduate School, Seoul, Korea; 5 Institute for Innovation in Digital Healthcare, Yonsei University, Seoul, Korea

**Keywords:** Atherosclerotic cardiovascular disease, Social network, Machine learning

## Abstract

**OBJECTIVES:**

Extensive evidence indicates that social factors play an essential role in explaining atherosclerotic cardiovascular disease (ASCVD). This study aimed to examine which social factors are associated with the estimated 10-year ASCVD risk score among male and female adults, incorporating both multifaceted social network components and conventional risk factors.

**METHODS:**

Using data from 4,368 middle-aged Korean adults, we explored factors most likely to explain ASCVD risk with interpretable machine learning algorithms. The ASCVD risk was determined using the 10-year ASCVD risk score, as calculated using pooled cohort equations. Social network components were assessed through the name generator module. A total of 52 variables were included in the model.

**RESULTS:**

For male participants (area under the receiver operating characteristic curve [AUC], 0.65), the average years known for network members contributed most to ASCVD risk prediction (mean Shapley additive explanations value, 0.31), followed by spouse’s education level (0.22), medical history with diagnosis (0.18), and snoring frequency (0.14). By contrast, for female participants (AUC, 0.60), medical history with diagnosis was the strongest predictor (0.47), followed by logged income (0.21), education level (0.19), and the average number of years known in network members (0.17).

**CONCLUSIONS:**

Several important social factors were associated with the ASCVD risk score in both male and female adults. However, longitudinal research is needed to determine whether these factors predict future ASCVD events.

## GRAPHICAL ABSTRACT

[Fig f5-epih-47-e2025052]

## Key Message

Among 4,368 Korean adults aged 40-64 years, we identified key social factors associated with the estimated 10-year ASCVD risk score in males and females. For males, the “average years known in network members” showed the highest contribution to risk prediction, whereas for females, “medical history with diagnosis” was the most influential feature. These findings suggest that the relative importance of social and health factors in explaining ASCVD risk varies by sex.

## INTRODUCTION

Atherosclerotic cardiovascular disease (ASCVD) is the leading cause of premature death worldwide [1]. In Korea, the combined mortality rate from ischemic heart disease and cerebrovascular disease rose from 103.6 per 100,000 in 2012 (52.5 and 51.1, respectively) to 115.4 in 2022 (65.8 and 49.6, respectively), according to national cause-of-death statistics [2]. Recent studies further show that the crude incidence of acute myocardial infarction and stroke increased from 2011 to 2019, followed by modest declines in 2020, highlighting the persistent cardiovascular burden [3,4]. To reduce and prevent ASCVD risk, prior research has emphasized the importance of lifestyle factors, particularly social relationships, which are critical for accessing necessary resources [5,6].

Social relationships—often conceptualized as a form of social capital enabling individuals to access benefits through networks—are a fundamental human need [5]. Empirical studies have consistently shown that social relationships are key determinants of health and longevity [7,8]. In particular, research on social networks, defined as “the web of social relationships that surround an individual” [8], has linked them to ASCVD risk. For instance, social isolation and poor-quality relationships have been associated with greater risks of myocardial infarction, atherosclerosis, autonomic dysregulation, and hypertension [9,10]. Central to these associations is social support, defined as either the perceived satisfaction from social interactions [11] or “the provision of assistance or comfort to others, typically to help them cope with biological, psychological, and social stressors” [12], which is shaped by network structures where resources are exchanged [9]. Consequently, individuals with stronger networks are more likely to access emotional, informational, and financial support, reducing stress and discouraging harmful behaviors [13]. Socioeconomic status (SES) also shapes network quality; higher income and spousal education are linked to lower ASCVD risk through healthier lifestyles and greater resource access [7,14,15].

Although prior studies have demonstrated the influence of social factors on ASCVD risk, relatively little research has investigated which specific social factors, including both social network components and health-related variables, are most important in explaining ASCVD risk among male and female adults. To evaluate the simultaneous effects of multiple factors and their relative importance, machine learning (ML) approaches can be highly useful. ML algorithms are particularly suited for addressing multicollinearity and capturing complex interactions without strict model assumptions [16,17]. Furthermore, interpretable ML methods, such as extreme gradient boosting (XGBoost) combined with Shapley additive explanations (SHAP) values, can provide clear insights into the importance of individual predictors. Accordingly, this study applies ML approaches to identify key social factors associated with ASCVD risk in male and female adults.

## MATERIALS AND METHODS

### Study population

This study used baseline data from the Cardiovascular and Metabolic Diseases Etiology Research Center (CMERC) cohort, which recruited participants from Seoul and surrounding areas to identify cardiovascular risk factors and develop predictive tools [18]. Eligible participants were community-dwelling Korean adults aged 30-64 years, with no recent history of cancer or cardiovascular disease (CVD), no current pregnancy, and no plans to relocate within 2 years. Between 2013 and 2018, baseline assessments were conducted at 2 research clinics: Yonsei University College of Medicine in Seoul (n=4,060) and Ajou University School of Medicine in Suwon (n=4,037).

Of the 8,097 participants enrolled, 1,210 individuals under age 40 were excluded to enable calculation of the 10-year risk of ASCVD using the pooled cohort equations (PCEs), described in the following section. An additional 2,519 participants were excluded due to missing data on explanatory variables. The final analytic sample included 4,368 participants, of whom 69% were females aged 40-64 years.

### Atherosclerotic cardiovascular disease risk score

The outcome variable, ASCVD risk, was measured using PCEs, which estimate the 10-year ASCVD risk score. The American College of Cardiology (ACC) and American Heart Association (AHA) recommend PCEs as a risk calculator, providing sex-specific and race-specific estimates of 10-year absolute risks of ASCVD events, including non-fatal myocardial infarction, fatal coronary heart disease, and non-fatal or fatal stroke [19].

In this study, the ASCVD risk score was calculated using the PCE for non-Hispanic Whites [20]. Predictor variables included self-reported age, sex, race, diabetes diagnosis, current smoking status, antihypertensive medication use, total cholesterol, high-density lipoprotein cholesterol, and systolic blood pressure. We then classified individuals into risk groups according to the 2019 ACC/AHA guidelines: low risk (<5.0%), borderline risk (5.0-7.5%), intermediate risk (7.5-20.0%), and high risk (≥20.0%) [19]. For the binary outcome variable, we applied a cutoff of 5%, coding participants as low risk (0) or at risk (1).

### Social network

The CMERC cohort applied a social network module known as the name generator. This instrument was originally developed for the General Social Survey in the United States to explore social dynamics related to government structures and personal connections, including equality, family, work, religion, environment, national identity, and citizenship [21]. A version adapted for the Korean context has been implemented in large-scale social surveys and has demonstrated strong validity in capturing key network properties relevant to health research [22].

Participants were asked to name their spouse (if applicable) and up to 5 individuals with whom they had discussed important matters in the past year. For each listed person, participants provided information on relationship type (e.g., spouse, parent, friend, provider) and demographics (age, sex, education, residence). They also reported relationship duration, frequency of communication (phone, text, or social media), face-to-face contact, emotional closeness, and ease of discussing health issues. Based on these responses, we constructed 42 social network variables across 4 domains: (1) network size and demographics, (2) interaction frequency and communication intensity, (3) network structure (triads, density, and mediation), and (4) spousal relationship dynamics [5,8]. A detailed description of social network, socioeconomic, and health-related variables is provided in Supplementary Material 1.

### Statistical analysis

We applied the least absolute shrinkage and selection operator (LASSO) and XGBoost to identify key factors associated with ASCVD risk and used SHAP values to assess feature importance. These ML algorithms are widely applied in cardiovascular research because of their distinct strengths [16,17]; detailed mechanisms and advantages are described in the Supplementary Material 2. LASSO regression was used for preliminary variable selection, as it simultaneously performs regularization and shrinks coefficients of less relevant or correlated variables to zero. XGBoost was then used to build predictive models based on the parsimonious set of predictors identified by LASSO. This process improves accuracy and robustness, as XGBoost often outperforms traditional methods and other ML algorithms, particularly for structured data [16]. To avoid redundancy and collinearity, variables used to calculate the ASCVD risk score were excluded from the ML models.

We first applied LASSO regression to reduce model parameters, selecting the optimal penalty term (λ) via cross-validation. Following this step, we divided the data into training (80%; n=1,095 for male, n=2,399 for female) and test (20%; n=274 for male, n=600 for female) datasets, performed hyperparameter tuning for XGBoost, and selected optimal values. We then constructed sex-specific XGBoost models and evaluated them with the test datasets, calculating the area under the receiver operating characteristic curve (AUC). Within each model, we identified key factors by examining their relative importance to ASCVD risk using SHAP values, which quantify each variable’s contribution to increasing or decreasing the predicted probability of risk. Important factors were reported separately for male and female and compared by sex.

For descriptive statistics, we compared socio-demographic characteristics, social network composition, comorbidities, and health-related behaviors between male and female participants. The Welch 2-sample t-test was used for continuous variables to account for unequal sample sizes, and the chi-square test was applied to categorical variables. Given consistent evidence of sex differences in social network structure and CVD [13,23], all analyses were stratified by sex. Continuous variables were mean-centered and rescaled to have comparable standard deviations. Analyses were conducted using R version 4.3.3 (R Foundation for Statistical Computing, Vienna, Austria), with statistical significance defined as p-value <0.05.

### Ethics statement

The CMERC cohort study was approved by the Institutional Review Boards of Severance Hospital, Yonsei University Health System, Seoul, Korea (No. 4-2013-0661), and Ajou University School of Medicine, Suwon, Korea (AJIRB-BMR-SUR-13-272). Written informed consent was obtained from all participants and stored securely in locked file cabinets.

## RESULTS

### Socio-demographic characteristics

A total of 4,368 participants, including 1,369 male and 2,999 female, were analyzed. As shown in Supplementary Material 3, male and female differed significantly in socio-demographic characteristics, with the exception of age. Regarding education, a higher proportion of male had completed high school or higher degrees compared with female (93.71 vs. 83.36%). A similar pattern was observed for spousal education, with a larger proportion of female reporting that their spouses held at least a high school degree compared with male (92.11 vs. 90.50%). Annual income was also higher among male than among female. In addition, a greater proportion of male were currently married compared with female (99.78 vs. 98.63%).

### Comorbidities and health-related behaviors

**Figure 1.**
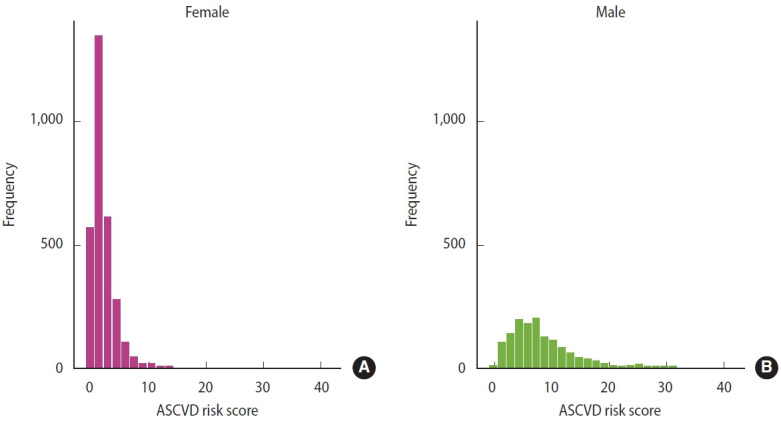
Distribution of atherosclerotic cardiovascular disease (ASCVD) risk score by sex. Histograms display the distribution of ASCVD risk scores among (A) female and (B) male participants. The ASCVD risk score was calculated using pooled cohort equations. The distribution for females is highly right-skewed, with most values concentrated below 5%, indicating a lower predicted risk overall. In contrast, male participants exhibit a broader distribution, with a higher proportion of individuals having risk scores above 5%.

Substantial sex differences were observed in comorbidities and health-related behaviors (Supplementary Material 3). Male adults had significantly higher prevalence of comorbidities and engaged more frequently in risk behaviors such as alcohol consumption and cigarette smoking compared with female adults. In terms of ASCVD risk, a score >5% was observed in 952 male (69.54%) and 221 female (7.37%), a difference that was statistically significant (p<0.001). This contrast is illustrated in Figure 1, where the peak frequency for female is concentrated at the very low end of ASCVD risk scores, whereas male display a broader distribution across the risk spectrum, though still with a higher proportion of low-risk individuals. These findings indicate that male are more likely than female to have elevated ASCVD risk scores.

### Network composition characteristics

Significant sex differences were found in 31 out of 42 social network variables. This suggests that male and female engage differently in social relationships, consistent with prior research showing that female are more likely to have confidence, form deeper bonds with network members, and maintain active connections with each member [24,25]. In Supplementary Material 3, female scored higher than male in measures such as close triads by affiliation, network density by affiliation, network density by communication frequency, total communication frequency (days), and average years known for network members. These findings indicate that female may be more likely than male to maintain close-knit inner circles of relationships [26].

Indicators of marital relationships further suggest that female engaged in more leisure activities with their spouses and relied on them more than male. This aligns with previous studies reporting that wives are more likely to disclose personal feelings and thoughts, while husbands tend to provide opinions or public-facing perspectives [27]. Thus, female appear to share more personal emotions and views with their husbands, while husbands offer feedback, fostering stronger emotional reliance within the marital bond [28].

#### LASSO regression

We calculated the minimum mean squared error (MSE) following 10-fold cross-validation (Supplementary Material 4). The penalty terms (λ) with the smallest MSE were -4.634 for male and -5.246 for female. These values were applied to the LASSO regression models to estimate coefficients for each variable. As presented in Supplementary Material 5, 21 variables were selected for male and 17 for female. Using the variables retained by LASSO regression, we evaluated the relative contribution of each feature to ASCVD risk prediction in the XGBoost model.

#### XGBoost model

**Figure 2.**
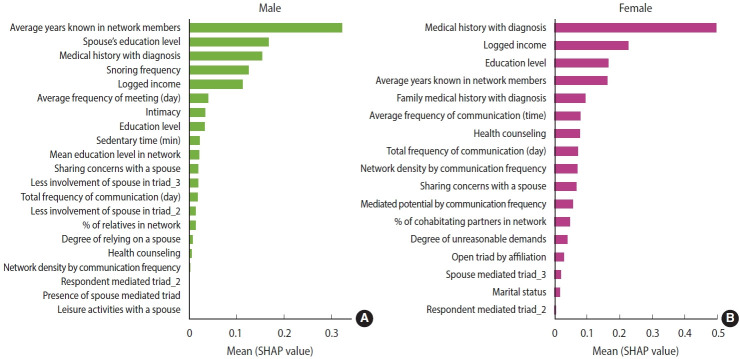
Feature importance of SHAP values extracted from XGBoost models trained to predict the ASCVD risk. The bar plots display the mean absolute SHAP values for each feature, representing their average contribution to the predicted ASCVD risk scores across the sample. (A) Male, and (B) female adults. Higher SHAP values indicate greater influence on model predictions. For males, the most influential predictors included average years known in network members, spouse’s education level, medical history with diagnosis, and snoring frequency. For females, the most important features were medical history with diagnosis, logged income, education level, and average years known in network members. SHAP, Shapley additive explanations; XGBoost, extreme gradient boosting; ASCVD, atherosclerotic cardiovascular disease.

To optimize model performance, hyperparameters for the XGBoost models were tuned, with results provided in Supplementary Material 6. The lowest root MSE values were selected as tuning parameters (Supplementary Materials 7 and 8). The final XGBoost models achieved AUC values of 0.65 for male and 0.60 for female. As shown in Figure 2, SHAP values derived from the XGBoost models highlighted the most important predictors of ASCVD risk, with results summarized in Table 1. Gray cells in Table 1 indicate overlapping factors between male and female.

**Figure 3.**
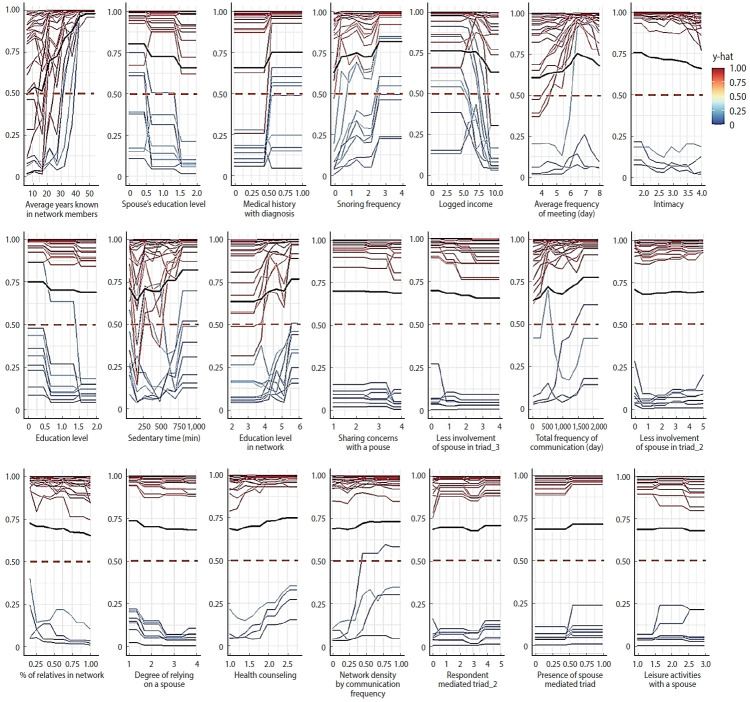
Probability of having the ASCVD risk among the important features in male. Each plot shows the predicted probability (y) of having elevated ASCVD risk across varying values of a given feature, as estimated by the XGBoost model. The color scale represents the predicted risk score (y-hat), ranging from low (blue) to high (red). The black line indicates the average predicted probability trend across individuals. Features are sorted by their relative importance in descending order (left to right, top to bottom), with the top-ranked variables (e.g., average years known in network members, spouse’s education level, and medical history with diagnosis) shown in the upper panels. ASCVD, atherosclerotic cardiovascular disease; XGBoost, extreme gradient boosting.

**Figure 4.**
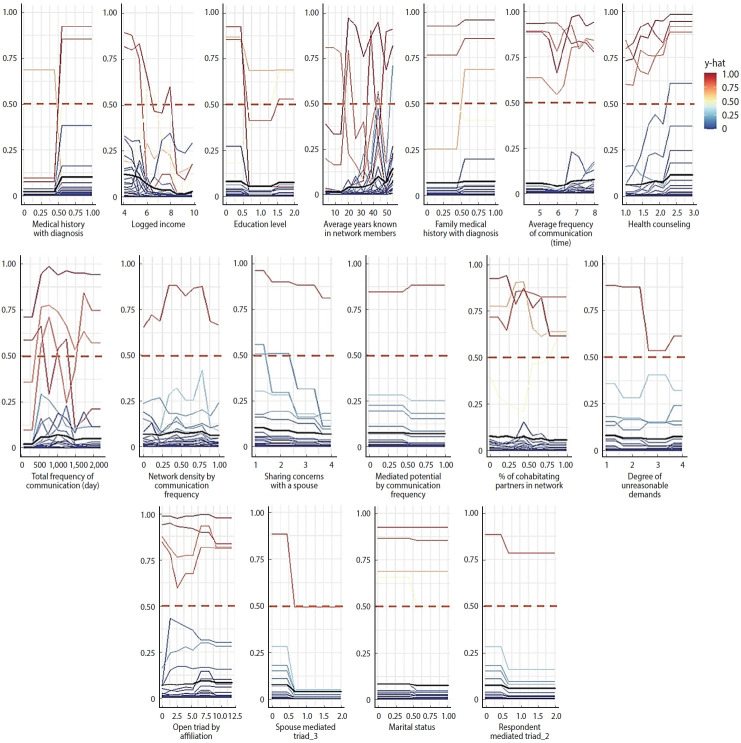
Probability of having the ASCVD risk among the important features in female. Each plot shows the predicted probability (y) of having elevated ASCVD risk across varying values of a given feature, as estimated by the XGBoost model. The color scale represents the predicted risk score (y-hat), ranging from low (blue) to high (red). The black line indicates the average predicted probability trend across individuals. Features are sorted by their relative importance in descending order (left to right, top to bottom), with the top-ranked variables (e.g., average years known in network members, spouse’s education level, and medical history with diagnosis) shown in the upper panels. ASCVD, atherosclerotic cardiovascular disease; XGBoost, extreme gradient boosting.

Figures 3 and 4 further illustrate the probability contributions of individual features to ASCVD risk predictions in male and female samples. The red lines at *y*-hat=1.00 indicate that in some cases, the predicted probability of the positive class (1=above 5%) reached its maximum of 100%.

## DISCUSSION

Using data from a community-based cohort of middle-aged Korean adults, we investigated sex-specific features of ASCVD risk. Consistent with previous research, male exhibited higher predicted ASCVD risk scores than female. However, this does not imply that female are inherently protected. Clinical studies have shown that hormonal changes—particularly those related to polycystic ovary syndrome (PCOS) and menopause—can elevate ASCVD risk in female. PCOS, characterized by excess androgen production, contributes to ovarian dysfunction and insulin resistance, while declining estrogen levels during menopause increase susceptibility to hypertension and ASCVD [29,30]. Thus, while male displayed a higher overall risk, interpreting this as a protective effect for female is misleading, especially given the impact of age-related hormonal changes.

We found that average years known for network members, medical history, logged income, and education level were common explanatory factors for ASCVD risk in both sexes. Notably, longer average relationship duration may indirectly reflect older age—a well-established risk factor for ASCVD—and may exert stronger effects in male than in premenopausal female [31]. Alternatively, longer relationships may signify more stable and emotionally rewarding ties, enhancing access to social support, health information, and positive behavioral influence [5,6]. However, relationship length does not necessarily ensure strong social ties, as variations in tie strength can affect support over time. This finding should therefore be interpreted cautiously, and future longitudinal studies should investigate how relationship quality and dynamics evolve across the life course.

Annual income and education are established social determinants of health, with extensive evidence demonstrating an inverse association between SES and ASCVD incidence [32,33]. Higher SES is linked to healthier behaviors—including smoking cessation, regular exercise, and better adherence to medical regimens—through improved behavioral control and long-term health planning [32]. Meta-analyses further indicate that individuals with higher SES benefit from better access to financial, informational, and social resources, including stronger support networks that reduce ASCVD risk [34]. For instance, Lee et al. [33] reported that older Korean adults with lower income had poorer medication adherence due to limited support and access to care, thereby increasing their ASCVD risk. These findings underscore the importance of SES as a determinant of ASCVD in both sexes.

Among social network components, we found that a greater average frequency of meetings and communication was associated with higher ASCVD risk. This counterintuitive result suggests that frequency alone may not confer health benefits; rather, the quality of interactions may be more decisive [7]. Frequent contact may at times reinforce harmful behaviors—such as smoking, alcohol use, or misinformation—rather than promote health [8,10]. For example, individuals with obese or smoking peers are more likely to adopt similar behaviors [35]. These findings emphasize that the health impact of social networks depends more on the quality than the quantity of interactions. Future research should examine the content of resources and activities exchanged during interactions, with particular attention to differences by sex and education level.

Spousal education emerged as an additional important factor for male but not for female. This aligns with research involving 37,618 married Israeli couples (aged 45-69), which showed that husbands’ CVD mortality was more strongly linked to wives’ education than to their own, while husbands’ education had little effect on wives’ outcomes [13]. Similarly, a Dutch study found higher coronary mortality among male with less-educated wives [36], and comparable findings have been reported elsewhere [13,37]. In contrast, a United States-based study reported opposite results, suggesting that husbands’ education was more important for wives’ self-rated health, while wives’ education had no significant effect [38]. Such discrepancies may reflect changing household roles and cultural variations in how education influences health within families.

Snoring frequency was the fourth most important ASCVD risk factor among male. Prior studies have found higher morbidity and mortality from ASCVD among male snorers [39,40]. Snoring, as a manifestation of sleep-disordered breathing, can reduce sleep quality and disrupt normal physiological function, thereby increasing ASCVD risk [39]. Habitual snorers also face greater risks of hypertension, coronary heart disease, and diabetes [40]. In this regard, snoring represents an important modifiable risk factor for ASCVD in male, suggesting that pharmacological interventions and lifestyle modifications may be beneficial in lowering risk.

Among female, sharing concerns with a spouse was identified as an important feature associated with ASCVD risk. Prior research has shown that spousal presence is linked to reduced ASCVD risk, likely due to the strong social, emotional, economic, and legal ties inherent in marital relationships [41]. Sharing concerns may foster emotional support and resource exchange, thereby enhancing well-being. Female lacking such support face higher risks of both fatal and non-fatal cardiovascular outcomes [42]. This form of spousal communication may reduce stress and improve health management [15], underscoring its protective role in lowering ASCVD risk among female.

Although family medical history (e.g., myocardial infarction, hypertension, stroke, or diabetes) showed only a modest effect in our model, it remains an important ASCVD risk factor, particularly for female. Studies have found that female with a family history of ASCVD exhibit heightened heart rate responses and reduced variability under stress, both linked to long-term cardiovascular risk [43]. A family history of premature myocardial infarction has also been associated with coronary artery calcification in female [43], and additional studies confirm its role as a significant risk factor [44,45]. This relationship likely reflects a combination of genetic predispositions affecting cholesterol, blood pressure, and metabolic regulation [44], as well as shared family behaviors—such as poor diet, smoking, and physical inactivity—that contribute to ASCVD risk [41]. While mechanisms are multifactorial, our findings reinforce the importance of family history in ASCVD risk assessment, particularly among female.

Despite its significant contribution to the existing literature, this study has several limitations. First, although we identified important factors related to ASCVD risk scores, we did not establish causal directions. Our outcome variable—the 10-year ASCVD risk—was estimated using the PCE, which relies on conventional clinical and behavioral risk factors within a cross-sectional design. As such, our findings reflect associations between social network variables and estimated risk rather than direct predictions of future ASCVD events. Second, discrepancies in feature importance between LASSO and XGBoost models were attributable to methodological differences. LASSO, as a regression-based model, does not capture feature interactions, whereas XGBoost, as a tree-based model, accounts for non-linear relationships and interactions. Third, the AUC values for the XGBoost models were below the commonly accepted threshold of 0.8-0.9. However, prior ASCVD prediction studies—particularly those employing ML algorithms—have generally reported more modest discriminative performance, with AUC values often ranging from 0.6 to 0.8, or even lower in some cases, due to imbalanced outcome distributions (reflecting the relatively low incidence of ASCVD events) within study datasets [e.g., 46,47].

Fourth, age and sex are strong predictors of ASCVD risk and are closely linked to social network characteristics. Given well-established sex differences in ASCVD incidence and risk profiles [48,49], we developed sex-specific models to identify important features within each group. However, direct comparisons of predictor importance between male and female remain limited by differences in baseline risk and social relationships. Similarly, age-related variation in network characteristics complicates efforts to isolate their independent effects. Although causal inference is restricted, our findings may nonetheless help identify individuals at higher risk based on social factors. Fifth, we employed the 10-year ASCVD risk score derived from the PCE, a validated tool widely used in cardiovascular risk prediction research across diverse populations [20,48,49]. However, because the PCE was originally developed in non-Asian cohorts, it may not be fully calibrated to Korean populations, raising the possibility of misestimation or bias in the observed associations. Our results should therefore be interpreted cautiously. To address this limitation, future studies may need to incorporate clinically measured outcomes—such as carotid intima-media thickness or incident cardiovascular events—to examine associations between social network characteristics and ASCVD risk. Finally, while the CMERC cohort included both general and high-risk individuals, providing diversity in health status, it was not nationally representative. This limitation may restrict the generalizability of our findings.

In conclusion, this cross-sectional study sought to identify key social factors associated with the estimated 10-year ASCVD risk score in male and female adults. We found several important social determinants, but future longitudinal research is needed to evaluate whether these factors can predict actual ASCVD events.

## Figures and Tables

**Figure f5-epih-47-e2025052:**
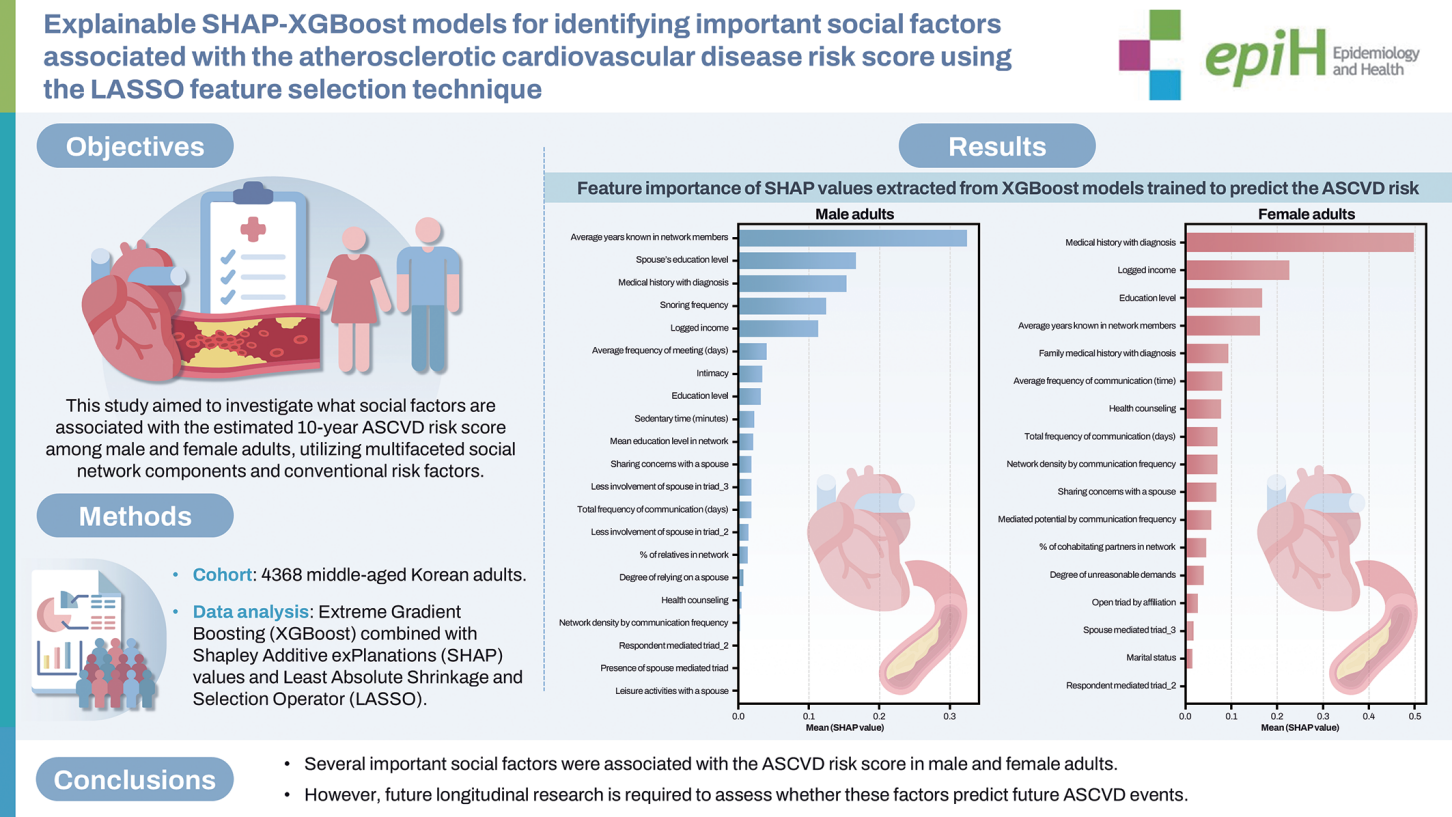


**Table 1.** Top 10 most important features

Importance	Male	Female
1	Average years known for network members	Medical history with diagnosis
2	Spouse’s education level	Logged income
3	Medical history with diagnosis	Education level
4	Snoring frequency	Average years known for network members
5	Logged income	Family medical history with diagnosis
6	Average frequency of meeting (day)	Average frequency of communication (time)
7	Intimacy	Health counseling
8	Education level	Total frequency of communication (day)
9	Sedentary time (min)	Network density by communication frequency
10	Education level in network	Sharing concerns with a spouse

## Supplementary materials

Supplementary Material 1. Description of study variables

Supplementary Material 2.

Supplementary Material 3. General characteristics of the study population (N = 4368)

Supplementary Material 4. Results of finding the optimal value of the penalty term (λ) in LASSO regression

Supplementary Material 5. LASSO regression coefficients of variables after adding the penalty term (N = 4368)

Supplementary Material 6. Hyperparameters used in XGBoost and their final chosen value

Supplementary Material 7. Results of hyperparameter values for fine-tuning the XGBoost algorithm’s behavior in male adults

Supplementary Material 8. Results of hyperparameter values for fine-tuning the XGBoost algorithm’s behavior in female adults
