# Supplementary material for: Explainable SHAP-XGBoost models for identifying important social factors associated with the atherosclerotic cardiovascular disease risk score using the LASSO feature selection technique
Source: Epidemiol Health. 2025 Sep 10;47:e2025052. doi: 10.4178/epih.e2025052 (PMC12869142; doi:10.4178/epih.e2025052)
Supplement: Supplementary Material 1. — Description of study variables [file epih-47-e2025052-Supplementary-1.docx]

Supplementary Material 1. Description of study variables

| Variable | Description |
| --- | --- |
| 1. Sociodemographic characteristics |  |
| Age | Respondent’s birthday (year) |
| Education level | 0=Less than high school, 1= High school graduate, 2=College/university or above |
| Spouse’s education level | 0=Less than high school, 1= High school graduate, 2=College/university or above |
| Logged income | Logged annual income |
| Marital status | 0=Single, 1=Currently married |
| 2. Comorbidities and health-related behaviors |  |
| TC (mg/dL) | Total cholesterol |
| HDL-C (mg/dL) | High-Density Lipoprotein Cholesterol |
| SBP (mmHg) | Average systolic blood pressure |
| Hypertension treatment | Currently taking medication for high blood pressure (0=No, 1=Yes) |
| Diabetes | Diagnosis of diabetes (0=No, 1=Yes) |
| Alcohol consumption | Current drinking status (0=No, 1=Yes) |
| Cigarette smoking | Current cigarette smoking status (0=No, 1=Yes) |
| Medical history with diagnosis | 0=No, 1=Yes |
| Family medical history with diagnosis | 0=No, 1=Yes |
| Depressive symptoms | 21 questions about depressive symptoms over the past 2 weeks (four-point Likert type scale, the Korean version of the Beck Depression Index II) |
| Sedentary time (minutes) | The amount of time spent in a day for sedentary behaviors during the past 7-day |
| Walking time (minutes) | The amount of time spent in a day for walking behaviors during the past 7-day |
| 3. Network composition characteristics |  |
| 1. Network size and demographics |  |
| Subjective network size | Number of people the respondent has talked to about important things in the past year (no limit). |
| Overall network size | Spouse + 5 people the respondent has talked to about important things + 1 important person (up to 7 people) |
| Network size | Spouse + 5 people the respondent has talked to about important things (up to 6 people). |
| Mean education level in network | Average education year of people in the network |
| % of same sex in network | Proportion of same sex in the network |
| % of cohabitating partners in network | Proportion of cohabitating partners |
| % of relatives in network | Proportion of relatives in the network |
| Average years known in network members | Average number of years the respondent has known people in the network |
| 1. Interaction frequency and communication intensity |  |
| Total frequency of communication (time) | Respondents were asked to rate how often they talked to each network member on an 8-point scale, ranging from ‘everyday’ to ‘less than once per year’ |
| Average frequency of communication (time) | Total frequency of communication ÷ Network size |
| Total frequency of communication (days) | Respondents was asked to rate how many days they talked to each network member on an 8-point scale, ranging from ‘everyday’ to ‘less than once per year.’ The scores were coded by  assigning the approximate number of times per year the respondent interacted with each member |
| Average frequency of communication (days) | Total frequency of communication by days ÷ Network size |
| Average frequency of meetings (days) | Total frequency of meetings ÷ Network size |
| Intimacy | Respondents were asked to rate how much they have intimacy with each network member on a 4-point scale, ranging from ‘strongly disagree to ‘strongly agree.’ The average score was calculated |
| Health counseling | Respondents were asked to rate the possibility of having health counseling when they have a health problem or make an important decision about their health on a 3-point scale, ranging from ‘Very likely’ to ‘Very unlikely.’ The average score was calculated |
| 1. Network structure (Triads, density, & mediation) |  |
| Closed triad by affiliation | Number of closed triadic relationships |
| Open triad by affiliation | Number of open triadic relationships |
| Network density by affiliation | The number of affiliations that exist between people in the network ÷ the total number of possible affiliations |
| Mediated potential by affiliation | The presence of people who are only affiliated with the respondent and not connected to others in the network (0=no, 1=yes) |
| Closed triad by communication frequency | Number of closed triadic relationships by communication (if the respondent has talked at least once every two weeks, it is considered connected) |
| Open triad by communication frequency | Number of open triadic relationships by communication (if the respondent has talked at least once every two weeks, it is considered connected) |
| Network density by communication frequency | The number of affiliations that exist between people in the network ÷ the total number of possible affiliations by communication (if the respondent has talked at least once every two weeks, it is considered connected) |
| Mediated potential by communication frequency | The presence of people who are only affiliated with the respondent and not connected to others in the network by communication (if the respondent has talked at least once every two weeks, it is considered connected) (0=no, 1=yes) |
| Closed triad by emotional closeness | Number of closed triadic relationships by emotional closeness (if the respondent has somewhat/a lot/very close relationships with network members, it is considered connected) |
| Open triad by emotional closeness | Number of open triadic relationships by emotional closeness (if the respondent has somewhat/a lot/very close relationships with network members, it is considered connected) |
| Network density by emotional closeness | The number of affiliations that exist between people in the network ÷ the total number of possible affiliations by emotional closeness (if the respondent has somewhat/a lot/very close relationships with network members, it is considered connected) |
| Mediated potential by emotional closeness | The presence of people who are only affiliated with the respondent and not connected to others in the network by emotional closeness (if the respondent has somewhat/a lot/very close relationships with network members, it is considered connected) (0=no, 1=yes) |
| Respondent mediated triad_1 | In a triadic relationship of respondent-spouse-third party, the spouse does not know the third party |
| Respondent mediated triad_2 | In a triadic relationship of respondent-spouse-third party, the spouse does not know the third party (the third party is a man) |
| Respondent mediated triad_3 | In a triadic relationship of respondent-spouse-third party, the spouse does not know the third party (the third party is a woman) |
| Less involvement of spouse in triad_1 | In a triadic relationship of the respondent-spouse-third party, the respondent communicates more with the third party than her or his spouse. |
| Less involvement of spouse in triad_2 | In a triadic relationship of the respondent-spouse-third party, the respondent communicates more with the third party than her or his spouse. (the third party is a man) |
| Less involvement of spouse in triad_3 | In a triadic relationship of the respondent-spouse-third party, the respondent communicates more with the third party than her or his spouse. (the third party is a woman) |
| 1. Spousal relationship dynamics |  |
| Spouse mediated triad_1 | In a triadic relationship of the respondent-spouse-third party, the spouse communicates more with the third party than her or his respondent |
| Spouse mediated triad_2 | In a triadic relationship of the respondent-spouse-third party, the spouse communicates more with the third party than her or his respondent (the third party is a man) |
| Spouse mediated triad_3 | In a triadic relationship of the respondent-spouse-third party, the spouse communicates more with the third party than her or his respondent (the third party is a woman) |
| Presence of spouse mediated triad | The situation where the spouse communicates more with the third party than her or his respondent (0=no, 1=yes) |
| Leisure activities with a spouse | 1=Do things together, 2=Do somethings together and some things separately, 3=Do different things separately (reverse coded) |
| Sharing concerns with a spouse | 1=I can't do it at all, 2=I can hardly do it, 3=I can do it sometimes, 4=I can do it often |
| Degree of relying on a spouse | 1=Never, 2=Rarely, 3=Sometimes, 4=Often |
| Degree of unreasonable demands | 1=Never, 2=Rarely, 3=Sometimes, 4=Often |
| Degree of blaming from a spouse | 1=Never, 2=Rarely, 3=Sometimes, 4=Often |
