# Supplementary material for: Explainable SHAP-XGBoost models for identifying important social factors associated with the atherosclerotic cardiovascular disease risk score using the LASSO feature selection technique
Source: Epidemiol Health. 2025 Sep 10;47:e2025052. doi: 10.4178/epih.e2025052 (PMC12869142; doi:10.4178/epih.e2025052)
Supplement: Supplementary Material 2. [file epih-47-e2025052-Supplementary-2.docx]

The LASSO regression is an extension of the ordinary least squares and the variable selection method. It selects the beta coefficients for minimizing the residual sum of squares (RSS). The formula is described below (1):


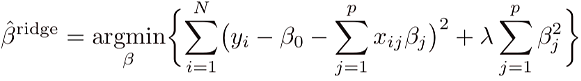
(1)

The formula shows that the penalty term, or lambda (λ), is to shrink the coefficients towards zero. Thus, increasing the lambda value results in a higher penalty, pushing more coefficients toward zero and leading to the reduction or elimination of specific features in the model, facilitating automatic feature selection. In contrast, lower lambda values diminish the penalty’s influence, preserving more features in the model; if the tuning parameter λ is set to zero, the LASSO will result in the least square regression. This shrinking method improves the interpretability of models by selecting relevant variables and promoting sparsity within the model; this can help avoid multicollinearity issues within datasets.^35^ As a result of this benefit, the LASSO regression has been extensively used in the big data domain, where selecting important variables from a multitude of options is crucial in predicting a response variable with low variance.

We used 10-fold cross-validation in the modeling process to select the optimal penalty term (λ). This method is crucial in determining the minimum mean squared error (MSE) score. Cross-validation involves randomly dividing the observations into k groups or folds of roughly equal size. The initial fold is selected as a test set, and the remaining k – 1 folds are designated as training sets. This ensures that all groups have an equal opportunity to be chosen as the test set. The cross-validation produces ten estimates of the MSE, and the mean of MSE calculates such estimates. The estimated value is then used to identify the optimal penalty term λ.

Unlike the LASSO regression, XGBoost is a tree-based ensemble ML method with unique features. It trains multiple weak leaders or models (high prediction errors) sequentially and ensemble them to produce a robust prediction model, called a boosting. This differs from a regular decision tree that depends on the largest information gain in the dataset. In this mechanism, every new learner is trained to predict the residual errors (the differences between the actual and predicted values) of the combined ensemble of previously trained models using gradient descent; this algorithm is called gradient boosting. XGBoost is an optimized and scalable version of gradient boosting due to its application of similarity score^[[1]](#footnote-1)^ and gain^1^ to decide the nodes of decision trees. With the application of these optimization techniques, XGBoost outperforms regular tree-based models with training speed and better predictive performance.

Setting hyperparameters in ML is essential for fine-tuning the algorithm’s behavior; thus, they must be established before training due to their impact on the model performance. XGBoost has various hyperparameters that need to be set. These include the degree of shrinkage for the feature weights to make the boost process more conservative (eta), the number of columns to be subsampled when constructing a tree (colsample), the max depth of a given decision tree (max_depth), subsample ratio of the training instances (sub_sample), the minimum child weight in a decision tree (min_child_wegith), and the minimum loss reduction required to make a partition (gamma). In order to enhance the precision of XGBoost, it is essential to optimize these hyperparameters.

The SHAP values introduced in 1951 within the cooperative game theory have recently been utilized as a machine learning tool for interpreting ensemble tree models, particularly XGBoost. This tool enhances clarity and transparency by making it easier to understand complex nonlinear models. SHAP values break down how input features (variables) influence the prediction of a machine learning model, quantifying the average contribution of a feature value when included in every possible combination of features. This approach helps us understand the impact of a feature’s particular value on the model’s prediction compared to a reference baseline value. The baseline value is the prediction the model would make without any information on feature values. This interpretability of SHAP values is a significant advantage, making it a commonly utilized technique for post-hoc explainability in determining feature attributions.

Before proceeding with the modeling of XGBoost, we randomly split the data to create a training set (80%) on which the model was built and a held-out test set (20%). This splitting of the data allows us to assess an algorithm’s performance by considering how well a machine-learning model can generalize to unseen data. The model performance is then evaluated using the receiver operating characteristic (ROC) scores that are calculated based on the area under the curve (AUC) that measures the ability of the model to distinguish between classes. Greater AUC values indicate a model’s improved ability to differentiate between positive and negative classes.

1. Similarity score = $\frac{{(\sum Residual)}^{2}}{\# of Residual+\lambda}$, and the Gain = similarity (left node) + similarity (right node) – similarity (root node). [↑](#footnote-ref-1)
