# Supplementary material for: Explainable SHAP-XGBoost models for identifying important social factors associated with the atherosclerotic cardiovascular disease risk score using the LASSO feature selection technique
Source: Epidemiol Health. 2025 Sep 10;47:e2025052. doi: 10.4178/epih.e2025052 (PMC12869142; doi:10.4178/epih.e2025052)
Supplement: Supplementary Material 3. — General characteristics of the study population (N = 4368) [file epih-47-e2025052-Supplementary-3.docx]

Supplementary Material 3. General characteristics of the study population (N = 4368)

| Variable | Male | Female | *p* value |
| --- | --- | --- | --- |
|  | (n = 1369) | (n = 2999) | $x^{2} or t$-test |
| 1. Sociodemographic characteristics |  |  |  |
| Age, year | 54.0 ± 6.58 | 53.8 ± 6.05 | 0.317 |
| Education level |  |  | <0.001 |
| Less than high school | 86 (6.29) | 499 (16.64) |  |
| High school graduate | 489 (35.72) | 1534 (51.15) |  |
| College/university or above | 794 (57.99) | 966 (32.21) |  |
| Spouse’s education level |  |  | <0.001 |
| Less than high school | 108 (7.89) | 285 (9.50) |  |
| High school graduate | 722 (52.74) | 1185 (39.52) |  |
| College/university or above | 539 (39.37) | 1529 (50.98) |  |
| Logged income | 6.90 ± 1.21 | 6.54 ± 1.08 | <0.001 |
| Marital status |  |  | <0.001 |
| Single | 3 (0.22) | 41 (1.37) |  |
| Currently married | 1366 (99.78) | 2958 (98.63) |  |
| 2. Comorbidities and health-related behaviors |  |  |  |
| TC (mg/dL) | 195 ± 32.0 | 200 ± 32.8 | <0.001 |
| HDL-C (mg/dL) | 50.1 ± 12.1 | 59.1 ± 13.7 | <0.001 |
| SBP (mmHg) | 125.7 ± 13.9 | 117.4 ± 14.6 | <0.001 |
| Hypertension treatment |  |  | <0.001 |
| No | 1049 (76.63) | 2545 (84.86) |  |
| Yes | 320 (23.37) | 454 (15.14) |  |
| Diabetes |  |  | <0.001 |
| No | 1183 (86.41) | 2766 (92.23) |  |
| Yes | 186 (13.59) | 233 (7.77) |  |
| Alcohol consumption |  |  | <0.001 |
| No | 187 (13.66) | 1129 (37.65) |  |
| Yes | 1182 (86.34) | 1870 (62.35) |  |
| Cigarette smoking |  |  | <0.001 |
| No | 966 (70.56) | 2972 (99.09) |  |
| Yes | 403 (29.44) | 27 (0.91) |  |
| Medical history with diagnosis |  |  | 0.233 |
| No | 718 (52.45) | 1513 (50.45) |  |
| Yes | 651 (47.55) | 1486 (49.55) |  |
| Family medical history with diagnosis |  |  | <0.05 |
| No | 573 (41.86) | 1156 (38.55) |  |
| Yes | 796 (58.14) | 1843 (61.45) |  |
| Depressive symptoms | 7.97 ± 6.17 | 9.38 ± 6.98 | <0.001 |
| Sedentary time (minutes) | 396 ± 196 | 334 ± 196 | <0.001 |
| Snoring frequency | 2.62 ± 1.07 | 2.22 ± 2.22 | <0.001 |
| ASCVD score | 8.19 ± 5.52 | 2.19 ± 1.92 | <0.001 |
| ASCVD score |  |  | <0.001 |
| <5% | 417 (30.46) | 2778 (92.63) |  |
| Above 5% | 952 (69.54) | 221 (7.37) |  |
| 3. Social network composition characteristics |  |  |  |
| Subjective network size | 6.18 ± 26.6 | 4.48 ± 5.23 | <0.001 |
| Overall network size | 4.60 ± 1.51 | 4.66 ± 1.48 | .183 |
| Network size | 3.88 ± 1.42 | 3.93 ± 1.40 | .285 |
| Education level in network | 4.51 ± 0.55 | 4.36 ± 0.58 | <0.001 |
| % of same sex in network | 0.61 ± 0.19 | 0.65 ± 1.7 | <0.001 |
| % of cohabitating partners in network | 0.33 ± 0.16 | 0.34 ± 0.18 | <0.05 |
| % of relatives in network | 0.46 ± 0.26 | 0.57 ± 0.28 | <0.001 |
| Average years known in network members | 27.1 ± 10.1 | 27.9 ± 9.69 | <0.05 |
| Total frequency of communication (time) | 26.3 ± 9.52 | 27.7 ± 9.69 | <0.001 |
| Average frequency of communication (time) | 6.84 ± 0.78 | 7.12 ± 0.66 | <0.001 |
| Total frequency of communication (days) | 790 ± 359 | 902 ± 381 | <0.001 |
| Average frequency of communication (days) | 212 ± 71.8 | 238 ± 73.9 | <0.001 |
| Average frequency of meeting (days) | 180 ± 74.1 | 187 ± 75.2 | <0.01 |
| Intimacy | 3.17 ± 0.53 | 3.12 ± 0.55 | <0.01 |
| Health counseling | 1.41 ± 0.42 | 1.28 ± 0.35 | <0.001 |
| Closed triad by affiliation | 4.95 ± 3.89 | 5.23 ± 4.06 | <0.05 |
| Open triad by affiliation | 1.64 ± 2.51 | 1.51 ± 2.26 | 0.09 |
| Network density by affiliation | 0.81 ± 0.25 | 0.83 ± 0.23 | <0.05 |
| Mediated potential by affiliation |  |  | 0.309 |
| No | 1239 (90.50) | 2744 (91.50) |  |
| Yes | 130 (9.50) | 255 (8.50) |  |
| Closed triad by communication frequency | 1.92 ± 2.52 | 2.06 ± 2.49 | 0.08 |
| Open triad by communication frequency | 4.67 ± 4.18 | 4.67 ± 4.08 | 0.971 |
| Network density by communication frequency | 0.29 ± 0.32 | 0.33 ± 0.33 | <0.001 |
| Mediated potential by communication frequency | 0.79 ± 0.41 | 0.75 ± 0.44 | <0.01 |
| Closed triad by emotional closeness | 3.64 ± 3.38 | 3.57 ± 3.33 | 0.524 |
| Open triad by emotional closeness | 2.96 ± 3.29 | 3.17 ± 3.33 | <0.05 |
| Network density by emotional closeness | 0.57 ± 0.35 | 0.56 ± 0.35 | 0.481 |
| Mediated potential by emotional closeness | 0.40 ± 0.49 | 0.43 ± 0.50 | 0.057 |
| Respondent mediated triad_1 | 0.29 ± 0.79 | 0.26 ± 0.67 | 0.096 |
| Respondent mediated triad_2 | 0.26 ± 0.72 | 0.02 ± 0.16 | <0.001 |
| Respondent mediated triad_3 | 0.03 ± 0.23 | 0.24 ± 0.64 | <0.001 |
| Less involvement of spouse in triad_1 | 2.74 ± 1.40 | 2.88 ± 1.40 | <0.01 |
| Less involvement of spouse in triad_2 | 2.41 ± 1.39 | 0.24 ± 0.55 | <0.001 |
| Less involvement of spouse in triad_3 | 0.32 ± 0.65 | 2.64 ± 1.38 | <0.001 |
| Spouse mediated triad_1 | 0.14 ± 0.45 | 0.05 ± 0.26 | <0.001 |
| Spouse mediated triad_2 | 0.08 ± 0.29 | 0.02 ± 0.13 | <0.001 |
| Spouse mediated triad_3 | 0.07 ± 0.28 | 0.03 ± 0.21 | <0.001 |
| Presence of spouse mediated triad |  |  | <0.001 |
| No | 1225 (89.48) | 2877 (95.93) |  |
| Yes | 144 (10.52) | 122 (4.07) |  |
| Leisure activities with a spouse | 1.80 ± 0.72 | 1.99 ± 0.81 | <0.001 |
| Sharing concerns with a spouse | 3.49 ± 0.72 | 3.45 ± 0.72 | 0.185 |
| Degree of relying on a spouse | 3.26 ± 0.79 | 3.49 ± 0.74 | <0.001 |
| Degree of unreasonable demands | 2.15 ± 0.84 | 2.14 ± 0.89 | 0.606 |
| Degree of blaming from a spouse | 2.19 ± 0.98 | 2.05 ± 0.96 | <0.001 |
