# Supplementary material for: Explainable SHAP-XGBoost models for identifying important social factors associated with the atherosclerotic cardiovascular disease risk score using the LASSO feature selection technique
Source: Epidemiol Health. 2025 Sep 10;47:e2025052. doi: 10.4178/epih.e2025052 (PMC12869142; doi:10.4178/epih.e2025052)
Supplement: Supplementary Material 4. — Results of finding the optimal value of the penalty term (λ) in LASSO regression [file epih-47-e2025052-Supplementary-4.docx]

Supplementary Material 4. Results of finding the optimal value of the penalty term (λ) in LASSO regression


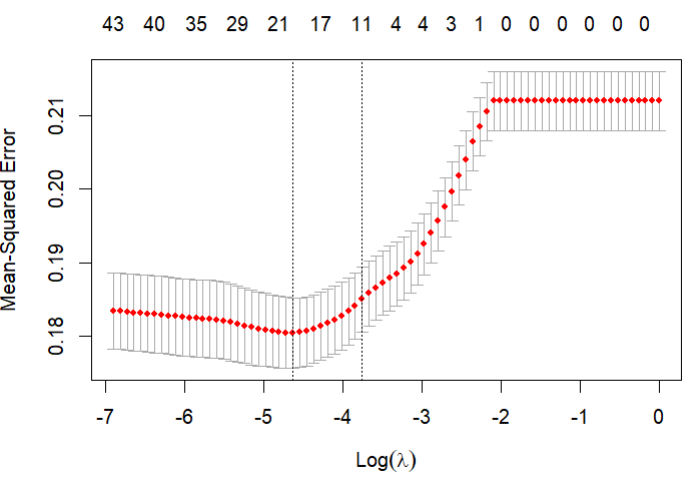

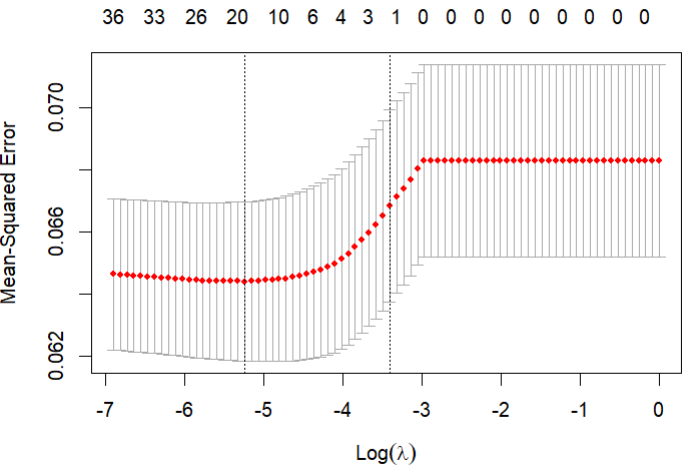


Note. The left figure is the result of males, and the right figure is female
