# Supplementary material for: Explainable SHAP-XGBoost models for identifying important social factors associated with the atherosclerotic cardiovascular disease risk score using the LASSO feature selection technique
Source: Epidemiol Health. 2025 Sep 10;47:e2025052. doi: 10.4178/epih.e2025052 (PMC12869142; doi:10.4178/epih.e2025052)
Supplement: Supplementary Material 5. — LASSO regression coefficients of variables after adding the penalty term (N = 4368) [file epih-47-e2025052-Supplementary-5.docx]

Supplementary Material 5. LASSO regression coefficients of variables after adding the penalty term (N = 4368)

| Male Adults | | |  | Female Adults | | |
| --- | --- | --- | --- | --- | --- | --- |
| No | Variable | Coefficient |  | No | Variable | Coefficient |
| 1 | Spouse’s education level | -0.103 |  | 1 | Marital status | -0.042 |
| 2 | % of relatives in network | -0.101 |  | 2 | Spouse mediated triad_3 | -0.034 |
| 3 | Intimacy | -0.058 |  | 3 | *Logged income* | -0.022 |
| 4 | *Logged income* | -0.032 |  | 4 | *Education level* | -0.018 |
| 5 | *Education level* | -0.030 |  | 5 | Respondent mediated triad_2 | -0.017 |
| 6 | Less involvement of spouse in triad_2 | -0.009 |  | 6 | % of cohabitating partners in network | -0.012 |
| 7 | *Sharing concerns with a spouse* | -0.003 |  | 7 | Mediated potential by communication frequency | -0.010 |
| 8 | Degree of relying on a spouse | -0.002 |  | 8 | *Sharing concerns with a spouse* | -0.008 |
| 9 | *Total frequency of communication (days)* | 0.0001 |  | 9 | Degree of unreasonable demands | -0.0008 |
| 10 | Sedentary time (minutes) | 0.0001 |  | 10 | *Total frequency of communication (days)* | 0.00001 |
| 11 | Snoring frequency | 0.0002 |  | 11 | Open triad by affiliation | 0.0001 |
| 12 | Leisure activities with a spouse | 0.001 |  | 12 | *Average years known in network members* | 0.002 |
| 13 | Respondent mediated triad_2 | 0.008 |  | 13 | Average frequency of communication (time) | 0.004 |
| 14 | Less involvement of spouse in triad_3 | 0.012 |  | 14 | Family medical history with diagnosis | 0.005 |
| 15 | *Average years known in network members* | 0.013 |  | 15 | *Health counseling* | 0.012 |
| 16 | *Health counseling* | 0.014 |  | 16 | *Network density by communication frequency* | 0.013 |
| 17 | *Presence of spouse mediated triad* | 0.016 |  | 17 | *Medical history with diagnosis* | 0.078 |
| 18 | *Network density by communication frequency* | 0.027 |  | 18 | Spouse’s education level | 0 |
| 19 | Average frequency of meeting (days) | 0.031 |  | 19 | % of relatives in network | 0 |
| 20 | Education level in network | 0.033 |  | 20 | Intimacy | 0 |
| 21 | *Medical history with diagnosis* | 0.109 |  | 21 | Less involvement of spouse in triad_3 | 0 |
| 22 | Marital status | 0 |  | 22 | Degree of relying on a spouse | 0 |
| 23 | Alcohol consumption | 0 |  | 23 | Sedentary time (minutes) | 0 |
| 24 | Family medical history with diagnosis | 0 |  | 24 | Snoring frequency | 0 |
| 25 | Depressive symptoms | 0 |  | 25 | Leisure activities with a spouse | 0 |
| 26 | Subjective network size | 0 |  | 26 | Less involvement of spouse in triad_2 | 0 |
| 27 | Overall network size | 0 |  | 27 | Average frequency of meeting (days) | 0 |
| 28 | Network size | 0 |  | 28 | Education level in network | 0 |
| 29 | % of same sex in network | 0 |  | 29 | Alcohol consumption | 0 |
| 30 | % of cohabitating partners in network | 0 |  | 30 | Depressive symptoms | 0 |
| 31 | Total frequency of communication (time) | 0 |  | 31 | Subjective network size | 0 |
| 32 | Average frequency of communication (time) | 0 |  | 32 | Overall network size | 0 |
| 33 | Average frequency of communication (days) | 0 |  | 33 | Network size | 0 |
| 34 | Closed triad by affiliation | 0 |  | 34 | % of same sex in network | 0 |
| 35 | Open triad by affiliation | 0 |  | 35 | Total frequency of communication (time) | 0 |
| 36 | Network density by affiliation | 0 |  | 36 | Average frequency of communication (days) | 0 |
| 37 | Mediated potential by affiliation | 0 |  | 37 | Closed triad by affiliation | 0 |
| 38 | Closed triad by communication frequency | 0 |  | 38 | Open triad by communication frequency | 0 |
| 39 | Open triad by communication frequency | 0 |  | 39 | Mediated potential by affiliation | 0 |
| 40 | Mediated potential by communication frequency | 0 |  | 40 | Closed triad by communication frequency | 0 |
| 41 | Closed triad by emotional closeness | 0 |  | 41 | Network density by affiliation | 0 |
| 42 | Open triad by emotional closeness | 0 |  | 42 | Closed triad by emotional closeness | 0 |
| 43 | Network density by emotional closeness | 0 |  | 43 | Open triad by emotional closeness | 0 |
| 44 | Mediated potential by emotional closeness | 0 |  | 44 | Network density by emotional closeness | 0 |
| 45 | Respondent mediated triad_1 | 0 |  | 45 | Mediated potential by emotional closeness | 0 |
| 46 | Respondent mediated triad_3 | 0 |  | 46 | Respondent mediated triad_1 | 0 |
| 47 | Less involvement of spouse in triad_1 | 0 |  | 47 | Respondent mediated triad_3 | 0 |
| 48 | Spouse mediated triad_1 | 0 |  | 48 | Less involvement of spouse in triad_1 | 0 |
| 49 | Spouse mediated triad_2 | 0 |  | 49 | Spouse mediated triad_2 | 0 |
| 50 | Spouse mediated triad_3 | 0 |  | 50 | Spouse mediated triad_1 | 0 |
| 51 | Degree of unreasonable demands | 0 |  | 51 | Presence of spouse mediated triad | 0 |
| 52 | Degree of blaming from a spouse | 0 |  | 52 | Degree of blaming from a spouse | 0 |
| *R*^2^ = 0.178 | |  |  | *R*^2^ = 0.071 | |  |
| n = 1369 | |  |  | n = 2999 | |  |
| λ = -4.634 | |  |  | λ = -5.246 | |  |
