# Supplementary material for: Explainable SHAP-XGBoost models for identifying important social factors associated with the atherosclerotic cardiovascular disease risk score using the LASSO feature selection technique
Source: Epidemiol Health. 2025 Sep 10;47:e2025052. doi: 10.4178/epih.e2025052 (PMC12869142; doi:10.4178/epih.e2025052)
Supplement: Supplementary Material 6. — Hyperparameters used in XGBoost and their final chosen value [file epih-47-e2025052-Supplementary-6.docx]

Supplementary Material 6. Hyperparameters used in XGBoost and their final chosen value

| Parameter | Value | RMSE |
| --- | --- | --- |
| Max_depth | 2 | 38.41 |
|  | 4 | 38.35 |
|  | 6 | 38.48 |
|  | 10 | 38.41 |
| Eta | 0.05 | 32.65 |
|  | 0.1 | 32.75 |
|  | 0.2 | 31.90 |
|  | 0.5 | 33.33 |
|  | 1 | 40.53 |
| Colsample | 0.33 | 31.26 |
|  | 0.66 | 32.65 |
|  | 0.5 | 32.29 |
|  | 1 | 31.83 |
| Gamma | 0.1 | 31.04 |
|  | 1 | 32.05 |
|  | 10 | 31.18 |
|  | 100 | 31.01 |
| Subsample | 0.25 | 58.06 |
|  | 0.5 | 59.74 |
|  | 0.75 | 64.62 |
|  | 1 | 58.46 |
| Min_child_weights | 1 | 58.06 |
|  | 10 | 59.74 |
|  | 100 | 64.62 |
|  | 400 | 58.46 |
