# Supplementary material for: Explainable SHAP-XGBoost models for identifying important social factors associated with the atherosclerotic cardiovascular disease risk score using the LASSO feature selection technique
Source: Epidemiol Health. 2025 Sep 10;47:e2025052. doi: 10.4178/epih.e2025052 (PMC12869142; doi:10.4178/epih.e2025052)

Supplementary Material 8. Results of hyperparameter values for fine-tuning the XGBoost algorithm’s behavior in female adults


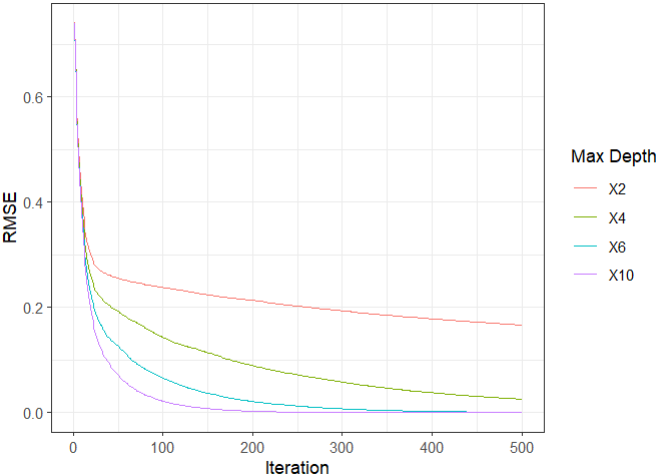

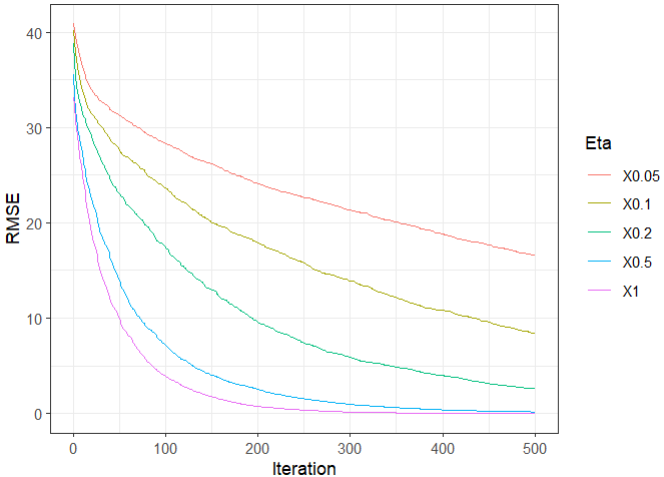

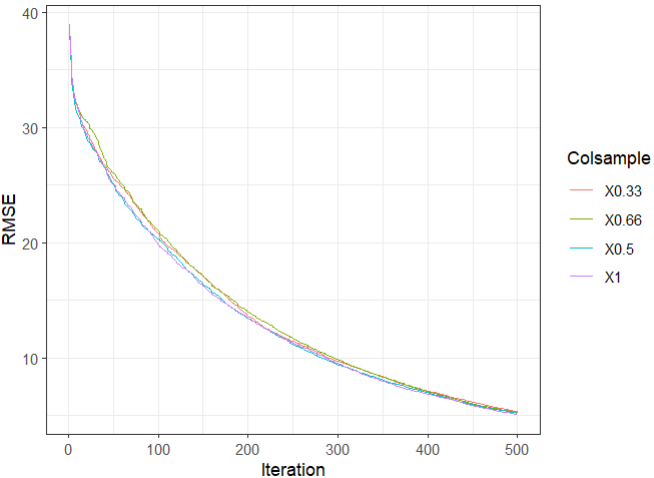


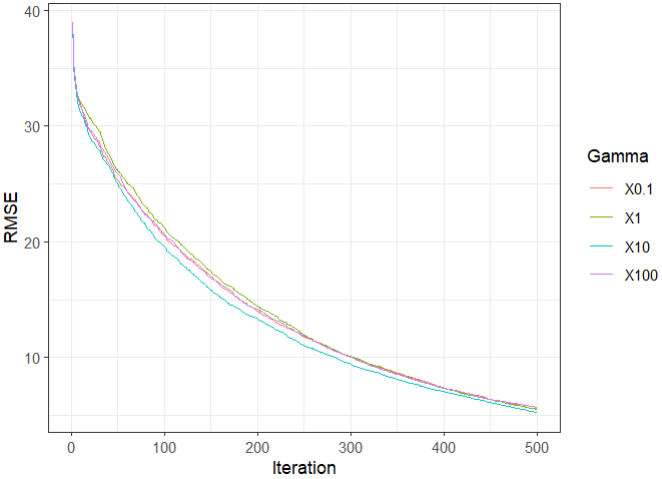

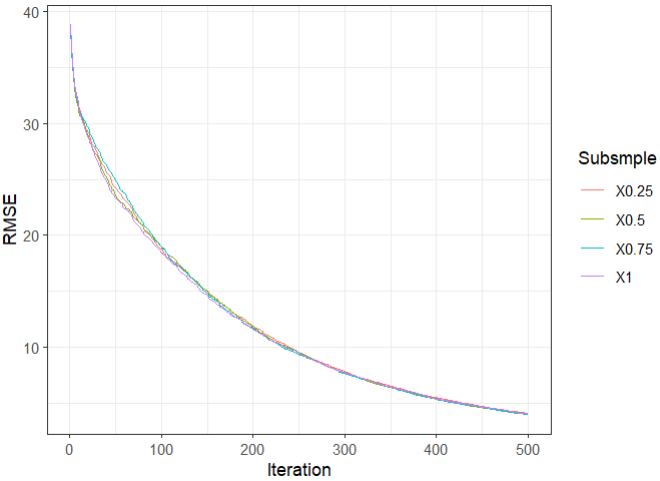

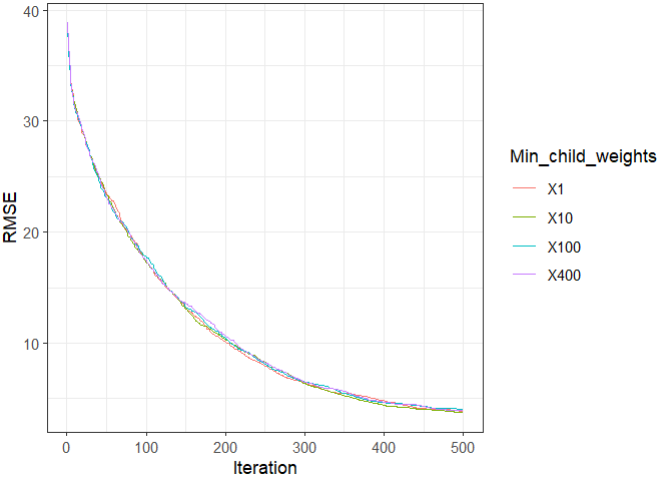

Supplement: Supplementary Material 8. — Results of hyperparameter values for fine-tuning the XGBoost algorithm’s behavior in female adults [file epih-47-e2025052-Supplementary-8.docx]
